# Full Genomic Analysis of New Variants of Porcine Reproductive and Respiratory Syndrome Virus Revealed Multiple Recombination Events Between Different Lineages and Sublineages

**DOI:** 10.3389/fvets.2020.00603

**Published:** 2020-09-10

**Authors:** Jinglong Wang, Siyuan Lin, Dongqun Quan, Hao Wang, Jiabin Huang, Yuxu Wang, Tongwei Ren, Kang Ouyang, Ying Chen, Weijian Huang, Tingrong Luo, Zuzhang Wei

**Affiliations:** Laboratory of Animal infectious Diseases and Molecular Immunology, College of Animal Science and Technology, Guangxi University, Nanning, China

**Keywords:** PRRSV, phylogenetic analysis, nsp2, deletion, recombination

## Abstract

Porcine reproductive and respiratory syndrome virus (PRRSV) has had a devastating impact on the pig industry in China, and monitoring its genetic diversity is important for epidemiological surveillance and understanding its evolution. Here, we determine the complete genome sequences of two PRRSV strains, GXYL1403 and GXNN1839. Comparative, phylogenetic, and recombination detection program analyses show that the two isolates are recombinant strains with large-fragment amino acid deletions in nsp2. GXYL1403 possesses a unique deletion region of 124 amino acids in nsp2, and GXNN1839 contains a deletion of 131 amino acids in nsp2 as compared with VR2332. Further analysis of the full-length sequence suggests that GXYL1403 is a natural recombinant between sublineages 8.1 (CH-1a like) and 8.3 (JXA1-like). The recombination site of GXYL1403 is located in nsp9–nsp12 (8961nt−11181nt). GXNN1839 is a natural recombinant between the lineage 5 (VR-2332-like) and lineage 1 (NADC30-like) strains. The recombination events occurred in nsp9 (7872nt-8162nt) and in ORF2 (12587nt−13282nt) in the genome of GXNN1839. These results provide new evidence that PRRSV strains circulating in the environment have undergone recombination among the different lineages or sublineages of field strains, and these add to our understanding of RNA combination events that occur in PRRSV.

## Introduction

Porcine reproductive and respiratory syndrome (PRRS) is an important infectious disease caused by porcine reproductive and respiratory syndrome virus (PRRSV), and it poses a serious threat to the global pig industry (1). PRRSV is a small, enveloped, positive-stranded RNA virus whose genome consists of at least 10 open reading frames (ORFs), namely ORF1a, ORF1b, ORF2a, ORF2b, ORF3, ORF4, ORF5, ORF5a, ORF6, and ORF7 (2). ORF1a and ORF1b are situated at the 5′ end of the genome and encode polyproteins that are self-cleaved to at least 16 non-structural proteins (nsps) by viral proteases (2, 3). ORF2a, ORF2b, ORF3, ORF4, ORF5, ORF5a, ORF6, and ORF7 are situated at the 3′ end of the genome and encode eight structural proteins, namely glycoprotein (GP) 2a, 2b, GP3, GP4, GP5, ORF5a protein, matrix protein (M), and nucleocapsid (N), respectively (3–5). PRRSV is divided into type 1 and 2 PRRSV genotypes, which only have about 60% nucleotide sequence identity (6, 7). Phylogenetic analyses of the ORF5 of type 2 PRRSV strains show that type 2 PRRSV could be divided into nine distinct lineages with each lineage containing several sublineages (8, 9). Type 2 PRRSV strains are prevalent in China and are distributed into four lineages and sublineages: lineages 1, 3, 5, and 8 are representatives of the NADC-30, QYYZ-like, VR-2332, and JXA1 strains, respectively (9). In 2006, a devastating outbreak of PRRS, caused by a highly pathogenic PRRSV (HP-PRRSV) representative with the JXA1 strain (sublineage 8.3) emerged in China, and this caused substantial economic losses for swine farming (10, 11). The lineage 3 PRRSV represented by the QYYZ strain emerged in 2010, and it is mainly circulating in southern China (12, 13). The NADC30-like PRRSV as represented by the WUH6 strain was first isolated in 2011 and is mainly found in northeast and central China (14–17).

The increasing diversity of the type 2 PRRSV is related to point mutations and genetic recombination among the field strains or in between the field and modified live vaccine (MLV) strains, which can lead to some new emerging antigenic variant strains (6, 18–24). In the present study, we identify two novel recombinant PRRSV strains circulating in swine farms in Guangxi, a province of southern China. We perform genome-wide sequence analysis of the two isolates and find new combination patterns in PRRSV evolution.

## Materials and Methods

### Cell Culture and Virus Isolation

Meat Animal Research Center-145 (MARC-145) cells and porcine alveolar macrophages (PAMs) were maintained in cell cultures as described in our previous study (25). Field samples (sera) were collected from clinically diseased pigs in Guangxi province, China. Samples were detected by RT-PCR using specific primers for the ORF5 gene of genotype 2 PRRSV (26). One positive sample was from 56 dying piglets exhibiting severe respiratory syndrome on a pig farm located in YuLing city, Guangxi Province, which has a total pig population of 823. One positive sample was from 18 pregnant sows suffering reproductive failure in a farm located in Nanning city, Guangxi Province in South China, which has a total sow population of 312. The positive samples were used for virus isolation using MARC-145 cells or PAMs. Cells were observed daily for the appearance of cytopathic effects (CPE). When 75% CPE was observed, the culture media were harvested and used for blinding passage in MARC-145 cells or PAMs. The PRRSV strain (GXYL1403) isolated from MARC-145 cells was then plaque-purified three times on MARC-145 cells for subsequent complete genome sequencing. The third passage of each virus strain was identified by RT-PCR and an indirect immunofluorescence assay (IFA).

### RT-PCR Amplification and Complete Genome Determination

The viral genomic RNA was extracted from PRRSV-infected PAMs or MARC-145 cells and reverse transcribed into cDNA using M-MLV reverse transcriptase (Takara) according to the manufacturer's instructions. PCR was performed to amplify the complete genomic sequences of PRRSV isolates GXYL1403 and GXNN1839 by using PRRSV-specific primers as described previously (27, 28). The PCR products were purified using an E.Z.N.A.TM Gel Extraction Kit (OMEGA, USA) and cloned into a pMD18-T vector (TaKaRa, Dalian, China) for nucleotide sequencing. The complete genomic sequences of PRRSV isolates were assembled using the SeqMan program of DNAstar software, version 7.0.

### Immunofluorescence Assay (IFA)

IFA was used to detect PRRSV N protein expression in PAMs and MARC-145 cells as described previously (25). At 72 h infection, the isolate-infected PAMs or MARC-145 cells were washed with phosphate-buffered saline (PBS) twice and fixed using cold methanol for 15 min at room temperature and then blocked with 3% BSA (fraction V bovine serum albumin) (Roche, Mannheim, Germany) for 30 min. The cells were treated with a monoclonal antibody (mAb) against PRRSV N protein (SR30-A) (Rural Technologies, Inc., Brookings, SD, USA) as the primary antibody and incubated for 2 h at 37°C. Then, the cells were washed five times with PBS and incubated with goat antirabbit IgG H&L (Alexa Fluor® 488, Abcam) for 1 h at 37°C. Following five further washes with PBS, the cells were observed under a fluorescence microscope.

### Sequence Comparison and Phylogenetic Analysis

Differences of sequences from the two isolates and reference strains from all over the world were analyzed and aligned using the MegAlign program in DNAstar 7.0 software (DNASTAR Inc., Madison, WI, USA). Phylogenetic trees, including the complete genomes and ORF5 from this study and the representative strains from China and other countries, were constructed by neighbor-joining in MEGA 6.0, using the maximum composite likelihood and bootstrap confidence values from 1,000 replicates. Detailed information on the PRRSV reference sequences is shown in Table 1.

**Table 1:** Information regarding the reference strains used in this study.

**No**.	**Virus strain**	**Accession no**.	**Country**	**Lineage**	**Phylogenetic tree**
1	NADC30	JN654459	American	1	GP5, Genome
2	MN184	EF442777	American	1	GP5, Genome
3	CHsx1401	KP861625	China	1	GP5, Genome
4	HNhx	KX766379	China	1	GP5, Genome
5	PRRSV0000008659	EU758687	American	2	GP5
6	PRRSV0000008973	EU758940	American	2	GP5
7	PRRSV0000031	DQ474791	American	2	GP5
8	GD-KP	KU978619	China	3	GP5, Genome
9	QYYZ	JQ308798	China	3	GP5, Genome
10	GM2	JN662424	China	3	GP5, Genome
11	Ibaraki08–5	AB546113	Japan	4	GP5
12	Miyagi08–2	AB546105	Japan	4	GP5
13	Miyagi08–3	AB546106	Japan	4	GP5
14	EDRD-1	AB288356	Japan	4	GP5, Genome
15	VR-23332	AY150564	American	5	GP5, Genome
16	NADC-8	AF396833	American	5	GP5
17	PA8	AH006184	Canada	5	GP5
18	NVSL-14	AF396839	American	6	GP5
19	Aichi N20	AB175715	Japan	7	GP5
20	Neb-1	EU755263	American	7	GP5, Genome
21	PrimePac	AF066384	American	7	GP5, Genome
22	CH-1a	AY032626	China	8.1	GP5, Genome
23	CH-1R	EU807840	China	8.1	GP5, Genome
24	HBJM2	EU399826	China	8.2	GP5
25	HBSZ	EU399825	China	8.2	GP5
26	JXA1	EF112445	China	8.3	GP5, Genome
27	JXA1 P80	FJ548853	China	8.3	GP5, Genome
28	JXwn06	EF641008	China	8.3	GP5, Genome
29	TJ	EU860248	China	8.3	GP5, Genome
30	JXZX2	HQ832215	China	8.4	GP5
31	HeN-2	FJ237419	China	8.5	GP5
32	AHW01	EU399828	China	8.5	GP5
33	GXLSN06–2012	KC618172	China	8.6	GP5
34	Yunnan-08	EU819086	China	8.6	GP5
35	HK1	KF287132	China	8.7	GP5, Genome
36	HK4	KF287134	China	8.7	GP5, Genome
37	JA-142	AF396842	American	9	GP5, Genome
38	SDSU73	AY656993	American	9	GP5, Genome
39	LV	M96262	Netherlands		GP5, Genome

### Recombination Analyses

The complete genomic sequences of GXYL1403 and GXNN1839 were compared with PRRSV reference strains JXA1, CH1a, VR-2332, and NADC30 using the Recombination Detection Program (RDP) v4.66 with six different algorithms (RDP, GENECONV, MaxChi, BootScan, SiScan, and 3Seq) (29). The results were presented using the RDP method. Recombinant events were confirmed by Bootscan analysis in Simplot software (v3.5.1, JHK University, Baltimore, MD, USA) with the default parameters. A series of phylogenetic trees based on each of the sequence regions were constructed to further identify these putative recombination events.

## Results

### Isolation and Identification of PRRSV Strains

Two PRRSV-positive sera were used to inoculate the MARC-145 cells and PAMs for PRRSV isolation. Typical CPE, characterized by cell fusion and shedding, was induced in both MARC-145 cells and PAMs 48 h post-inoculation (hpi) (data not shown). To confirm the isolation of the PRRSV strains, IFA was conducted using a mAb against PRRSV N (SR-30A). PAMs and MARC-145 cells infected with PRRSV isolates reacted with the specific antibody against the PRRSV N protein (data not shown). These results indicate successful isolation of the infectious PRRSV strains. These PRRSV isolates were designated as GXYL1403 and GXNN1839, respectively.

### Genomic Characteristics and Phylogenetic Analysis of the Two PRRSV Isolates

The full-length genomes of GXYL1403 and GXNN1839 were determined and submitted to the GenBank database with accession numbers MN660069 and MN660070. The entire genomes of GXYL1403 and GXNN1839 are 15,018 and 15,019 nucleotides in length, respectively, excluding the poly (A) tails as shown in Tables 2, 3. The nucleotide similarities of GXYL1403 with PRRSV representative strains NADC30, VR-2332, QYYZ, CH-1a, and JXA1 were 82.5, 88.8, 87.2, 94.2, and 98.0%, respectively, with only 60.6% nucleotide similarity with the type 1 PRRSV LV strain (Table 2). The nucleotide similarities of GXNN1839 with NADC30, VR-2332, QYYZ, CH-1a, and JXA1 were 92.2, 84.5, 81.9, 83.6, and 82.9%, respectively, with only 59.8% nucleotide similarity with the LV strain (Table 3). This indicates that the GXYL1403 and GXNN1839 strains belong to type 2 PRRSV. Each region of the GXYL1403 and GXNN1839 genomes were further compared with those of the reference strains as shown in Tables 2, 3. The results show that GXYL1403 exhibits the highest nucleotide similarity (92.8–100%) and amino acid similarity (93.4–100%) with JXA1. The 5′UTR and nsp8-nsp12 of GXYL1403 share the highest nucleotide similarity with that of CH-1a. The other regions of GXYL1403 exhibit the highest nucleotide and amino acid similarities with those of JXA1. GXNN1839 shares the highest nucleotide (86.4–97.9%) and amino acid (83.9–98.9%) similarities with the NADC30 strain (Table 3). ORF2–ORF4 of GXNN1839 share higher (92.6–96.5%) nucleotide and amino acid similarities (93–96.9%) with VR-2332 but exhibit lower nucleotide identity with the other reference strains. The other regions of the GXNN1839 genome exhibit the highest nucleotide and amino acid similarities with NADC30. These results indicate that GXYL1403 and GXNN1839 may be made up of mosaic isolates.

**Table 2:** Nucleotide and amino acid identities of different regions of GXYL1403 compared with other PRRSV reference strains.

**Genomic region**	**Pairwise % identity (nt/aa)**					
	**NADC30**	**VR-2332**	**QYYZ**	**CH-1a**	**JXA1**	**Lelystad virus**
Complete genome	82.5	88.8	87.3	94.2	98.0	60.6
5′UTR	90.6	92.6	95.8	94.9	90.8	62.2
Nsp1	83.8/85.1	87.9/89.0	87.7/89.6	92.6/91.4	96.8/96.1	57.6/53.0
Nsp2	68.6/58.5	84.0/79.0	83.4/78.6	90.8/86.4	97.0/96.1	34.8/29.5
Nsp3	82.5/90.4	90.7/94.6	82.1/88.6	95.1/98.2	98.9/99.1	59.6/58.9
Nsp4	83.5/92.6	89.7/93.1	84.2/91.7	95.4/95.6	99.0/98.5	62.7/63.1
Nsp5	86.5/91.2	89.4/92.4	82.4/90.0	94.3/94.7	98.8/100	64.0/71.8
Nsp6	93.8/100	95.8/93.8	97.9/100	97.9/100	100/100	68.8/75.0
Nsp7	81.6/83.4	88.5/89.2	92.8/94.6	95.0/96.1	98.6/99.2	57.7/46.5
Nsp8	88.1/91.2	95.6/97.8	94.1/97.8	99.3/97.8	98.5/97.8	63.7/66.7
Nsp9	86.2/93.1	91.5/97.7	90.8/97.0	95.4/98.1	97.9/99.1	67.2/75.0
Nsp10	83.7/87.2	89.3/96.5	89.4/97.0	93.1/97.7	96.7/98.9	60.4/64.5
Nsp11	89.3/96.4	89.4/95.5	87.5/94.6	96.2/98.7	94.5/93.4	66.1/77.2
Nsp12	89.4/71.9	88.9/70.6	86.8/62.7	95.4/88.8	92.8/93.6	44.1/45.8
ORF2	84.0/83.1	91.3/92.0	89.0/88.7	94.9/97.9	97.6/96.4	65.4/62.4
ORF3	82.6/80.8	89.0/87.1	90.7/87.8	95.9/93.3	99.1/97.6	65.2/57.1
ORF4	85.3/87.1	89.9/91.1	93.7/95.5	96.5/98.3	96.5/96.6	66.3/72.1
ORF5	84.1/84.5	88.4/87.1	83.1/80.6	94.5/91.5	99.2/98.5	64.4/57.4
ORF6	89.0/93.7	95.0/97.7	90.5/96.6	97.1/97.7	99.6/100	69.2/80.5
ORF7	90.3/90.1	92.7/93.5	89.0/91.1	96.5/96.0	99.2/99.2	69.2/63.6
3′UTR	89	92.6	90.0	92.7	95.2	65.4

**Table 3:** Nucleotide and amino acid identities of different regions of GXNN1839 compared with other type 2 PRRSV reference strains.

**Genomic region**	**Pairwise % identity (nt/aa)**					
	**NADC30**	**VR-2332**	**QYYZ**	**CH-1a**	**JXA1**	**Lelystad virus**
Complete genome	92.2	84.5	81.9	83.9	82.9	59.8
5′UTR	93.6	92.6	87.9	94.9	90.8	62.4
Nsp1	92.0/90.1	83.9/85.4	80.5/80.7	85.6/85.1	8771.7/81.5	56.9/53.8
Nsp2	89.6/83.9	76.6/68.6	72.7/63.3	77.7/69.9	73.2/64.6	33.3/29.9
Nsp3	90.9/92.8	84.3/94.1	80.0/86.3	83.3/90.8	81.2/93.6	60.2/57.5
Nsp4	93.6/97.5	85.5/93.1	83.7/91.7	84.2/92.2	84.6/93.6	61.2/61.6
Nsp5	92.2/92.9	87.5/89.4	81.6/89.4	86.2/92.9	85.5/91.8	63.5/70.0
Nsp6	89.6/93.8	87.5/100	85.4/93.8	89.6/90.8	87.5/93.8	72.9/81.2
Nsp7	92.3/90.3	84.0/85.7	79.5/83.4	82.8/85.7	80.4/83.4	55.2/48.0
Nsp8	91.1/98.9	88.1/88.9	83.7/88.9	84.4/88.9	85.9/86.7	60.0/68.9
Nsp9	92.9/96.9	87.8/96.6	85.1/95.2	86.6/86.8	86.4/88.9	67.8/74.2
Nsp10	93.8/97.7	85.6/94.8	83.9/93.4	84.7/85.5	84.2/86.9	60.8/64.5
Nsp11	94.6/96.0	87.2/95.1	86.0/94.2	87.7/86.4	89.4/92.9	65.8/76.3
Nsp12	93.7/83.7	87.0/90.2	84.2/90.8	88.0/90.8	88.0/92.2	44.3/42.7
ORF2	91.2/92.4	96.5/96.9	83.7/84.8	85.5/87.2	84.4/85.6	67.5/60.4
ORF3	92.6/92.7	94.8/93.0	85.1/82.7	87.5/79.6	86.0/79.2	66.0/57.5
ORF4	86.40/87.8	92.6/93.2	83.4/84.9	85.8/89.4	84.0/89.4	67.8/68.7
ORF5	93.0/95.0	87.0/85.6	84.8/83.0	86.7/87.6	86.7/87.6	61.9/53.3
ORF6	97.7/96.1	89.9/91.5	88.8/92.0	88.4/88.0	89.3/88.1	70.782.8
ORF7	94.9/97.4	90.9/92.4	86.6/88.7	90.1/93.5	89./91.9	65.8/60.3
3′UTR	98	92.6	85.7	87.8	89.1	64.9

**Figure 1:**
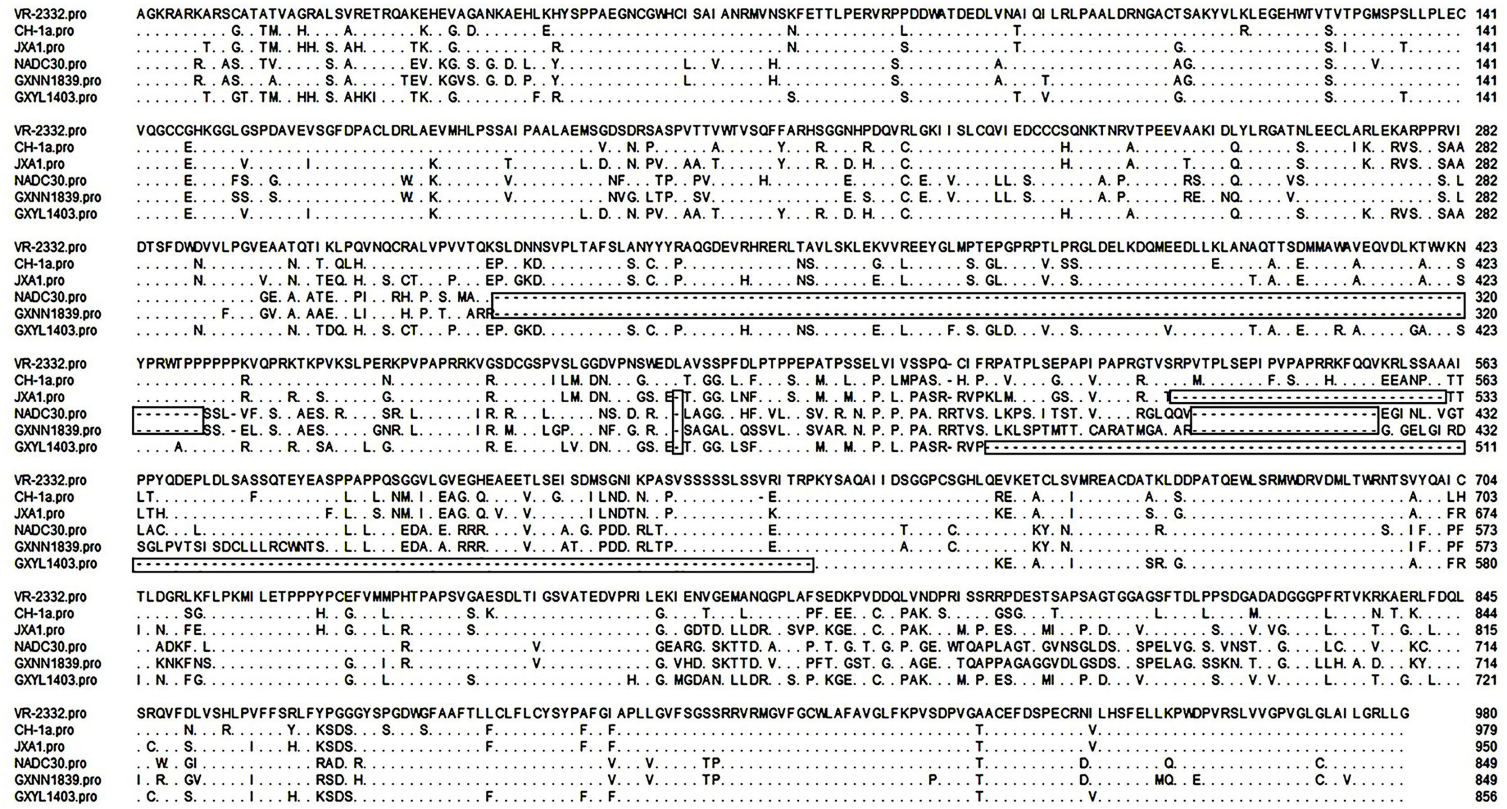
Identification of PRRSV strains with amino acid deletion in nsp2. Multiple alignments of nsp2 were performed by Clustal W. The sequence of VR-2332 is shown on the top; the residues conserved with it are hidden. The deleted amino acids are labeled with boxes.

Nsp2 is one of the most heterogeneous proteins in PRRSV and contains different patterns of amino acid insertions and deletions. Compared with VR-2332, GXYL1403 contains a discontinuous 124-amino acid deletion in the hypervariable region (HVR) of nsp2. GXNN1839 has a discontinuous 131-amino acid deletion in the HVR of nsp2, which has the same amino acid deletion pattern as the NADC30-like strain (Figure 1). This data suggest that novel PRRSV strains with amino acid deletions in nsp2 are emerging.

**Figure 2:**
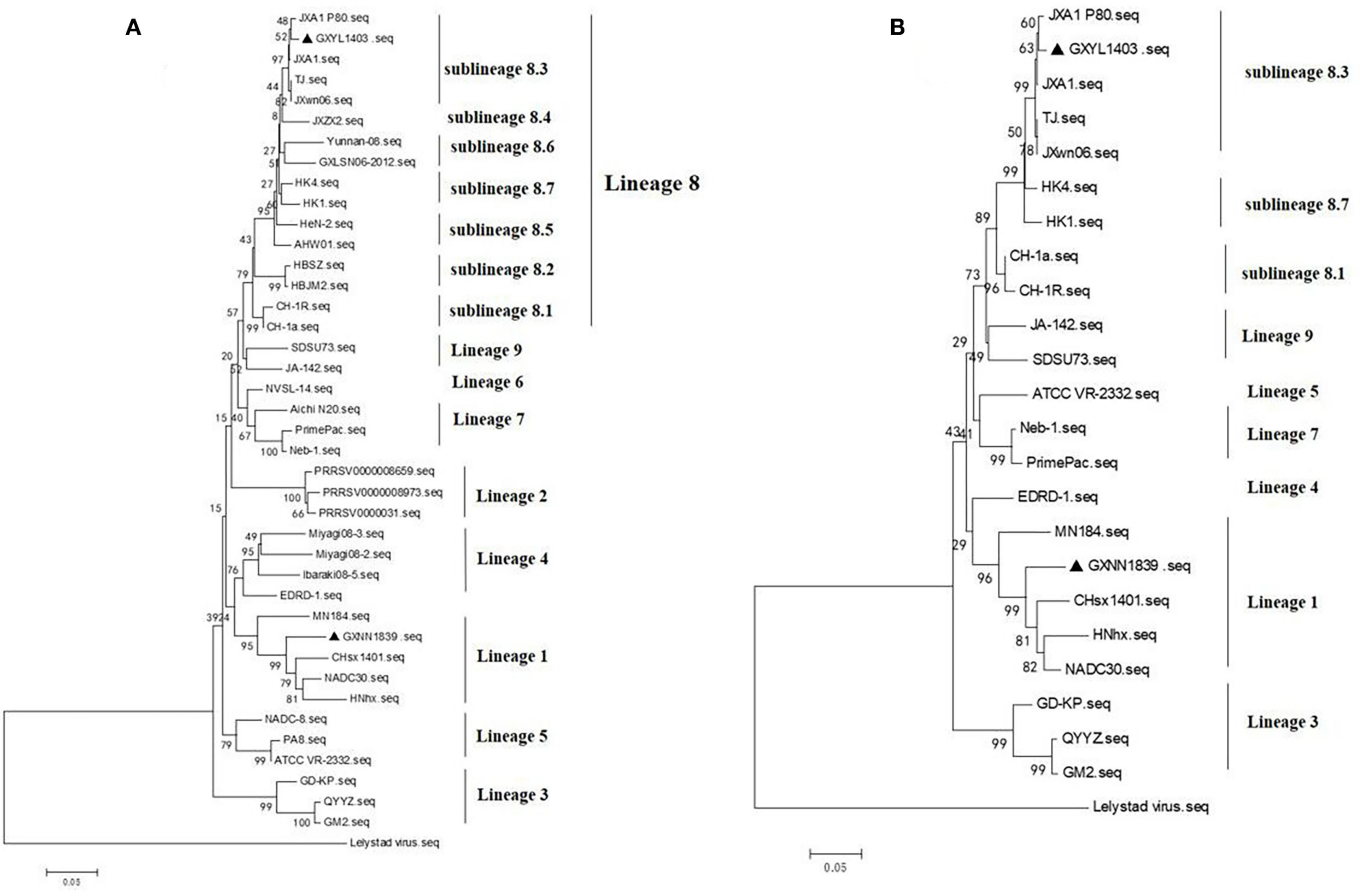
**(A)** Phylogenetic tree based on ORF5 sequences of isolates from this study and reference ORF5 sequences from PRRSV strains originating from China and other countries. The phylogenetic tree was constructed using the neighbor-joining algorithm based on the *p*-distance model. The PRRSV ORF5 sequences collected in this study are marked with black triangles. **(B)** Phylogenetic tree based on full-length genomic sequences of isolates. The GXYL1403 and GXNN1839 in this study and PRRSV reference strains are available in GenBank. The strains isolated in this study are labeled with black triangles.

The phylogenetic tree based on GP5 shows that the type 2 PRRSV strains can be classified into nine different lineages. GXYL1403 belongs to sublineage 8.3, which is represented by the reference strain JXA1. GXNN1839 belongs to lineage 3, which is represented by the reference strain NADC30 (Figure 2A). The phylogenetic tree based on the complete genome also shows that GXYL1403 belongs to sublineage 8.3, and GXNN1839 belongs to lineage 3 (Figure 2B).

### Recombination Analysis

**Figure 3:**
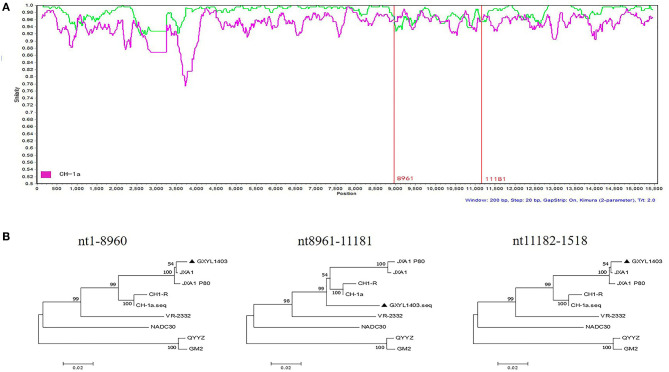
Identification of potential recombination events in the genome of the GXYL1403 strain using RDP3.44. **(A)** Comparisons of genetic similarities between recombinant and parental strains were made by SimPlot. The complete genome of GXYL1403 was chosen as the query sequence. The y-axis indicates the percentage similarity between the parental sequences and the query sequence. The red real diagram indicates the recombination breakpoints. **(B)** Phylogenetic trees of different regions in the PRRSV GXYL1403 genome were analyzed by RDP3.44 with UPGMA parsimony. Three trees were generated by the recombinant region (position: nt8961–nt11181) and the non-recombinant regions (position: nt1–8960 and nt11182–1518).

**Figure 4:**
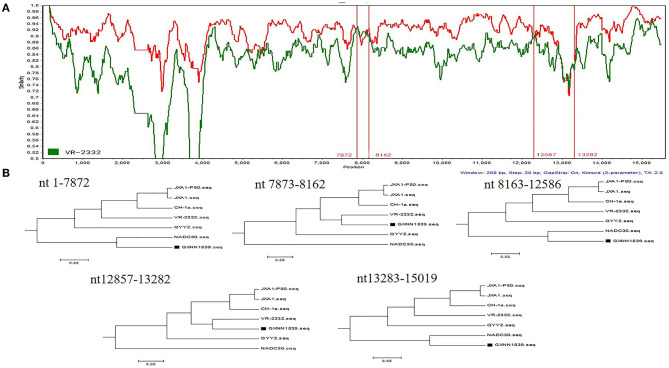
Identification of potential recombination events in the genome of the GXNN1839 strain using RDP3.44. **(A)** Comparisons of genetic similarities between recombinant and parental strains were made by SimPlot. The complete genome of GXNN1839 was chosen as the query sequence. The y-axis indicates the percentage similarity between the parental sequences and the query sequence. The red real diagram indicates the recombination breakpoints. **(B)** Phylogenetic trees of different regions in the GXYL1403 genome were analyzed by RDP3.44 with UPGMA parsimony. Five trees were generated by the recombinant regions (position: nt7873–8162 and nt12857–13282) and the non-recombinant regions (position: nt1–7872, nt8163–12586, and nt13283–15019).

To determine the recombinant events in the genomes of the GXYL1403 and GXNN1839 isolates, we performed recombinant detection using RDP4 and SimPlot by comparing GXYL1403 and GXNN1839 with the representative PRRSV strains from different lineages in the phylogenetic tree, including strains from lineage 1 (NADC30), lineage 3 (QYYZ), lineage 5 (VR2332), and lineage 8 (CH-1a and JX1A). The results show that putatively recombinant breakpoints located at positions 8961nt−11181nt in the GXYL1403 genome were detected with a high degree of reliability (Table 4). The breakpoints in the GXYL1403 genome separated its genome into three regions (Figure 3A). The phylogenetic trees also paralleled and confirmed the recombinant events as shown in Figure 3B. The region 1nt−8961nt is closely genetically related to the JXA1-like strain. The region 8961nt−11181nt is closely genetically related to the CH-1a-like strain, and the region 11182nt−5018nt is closely related to the JXA1-like strain (Figure 3B). It was suggested that GXYL1403 is a natural recombinant between sublineage 8.1 (CH-1a-like) and sublineage 8.3 (JXA1-like). Two putatively major recombinant breakpoints in the GXNN1839 genome were detected with high reliability, and they are located at the nsp9 coding region (7872nt−8162nt) and the ORF2 coding region (12587nt−13282nt), respectively (Table 4). The breakpoints in the GXNN1839 genome separated its genome into five regions (Figure 4A). The phylogenetic trees also paralleled and confirmed the recombinant events as shown in Figure 4B. This suggests that GXNN1839 is a natural recombinant between linage 5 (VR-2332-like) and lineage 1 (NADC30-like).

**Table 4:** Recombination breakpoints identified in GXYL1403, GXNN1839, and associated parental strains of PRRSV.

**Isolate**	**Breakpoints**		**Major parent**	**Minor parent**	***p*****-Value of the detection methods**						
	**Beginning**	**Ending**			**RDP**	**GENECONV**	**BootScan**	**MaxChi**	**Chimera**	**SiScan**	**3Seq**
GXNN1839	7,872	8,162	NADC30	VR-2332	3.373 × 10^−11^	3.971 × 10^−13^	2.189 × 10^−17^	1.190 × 10^−14^	2.497 × 10^−15^	3.534 × 10^−16^	1.545 × 10^−14^
	12,587	13,282	NADC30	VR-2332	5.253 × 10^−26^	6.773 × 10^−21^	1.725 × 10^−21^	1.316 × 10^−11^	1.655 × 10^−12^	2.546 × 10^−18^	2.22 × 10^−15^
GXYL1403	8,961	11,181	JXA1	CH-1a	3.408 × 10^−18^	6.05 × 10^−23^	2.648 × 10^−16^	1.146 × 10^−24^	2.228 × 10^−17^	3.782 × 10^−14^	1.515 × 10^−17^

## Discussion

PRRSV has had a large impact on the Chinese swine industry since its initial outbreak in China in 1996. In 2006, a large outbreak of PRRS caused by HP-PRRSV (10, 11, 26) emerged in most areas of China and caused major economic losses for swine farming. With the emergence of lineages 3 and 1 PRRSV in some areas in China in 2010 and 2015 (12, 14–17), respectively, the genetic diversity of RPRSV has significantly expanded and has further complicated vaccine development. In the present study, we isolated two PRRSV strains (GXYL1403 and GXNN1839) and determined their complete genome sequences. Consistent with previous studies, phylogenetic trees based on GP5 show type 2 PRRSV strains isolated in China are divided into four lineages (1, 3, 5, and 8) (9). The GXYL1403 and GXNN1839 strains reported in this study are grouped into lineages 1 and 3, respectively. The nsp2 is the most heterogeneous protein in PRRSV (26, 30, 31). Compared to strain VR-2332, the nsp2 of the two isolates in this study contain different amino acid deletions, which suggest that novel PRRSV strains with amino acid deletions in nsp2 are emerging.

Recombination plays a critical role in the genetic evolution of PRRSV and presents great challenges for the prevention and control of the disease it causes. Recombination can occur during the virus-replication process. It has been shown that there are different patterns of recombination among the different lineages and sublineages of PRRSV strains (14, 32–39). In the present study, whole genome sequence comparison and recombination analysis reveals that the two isolates (GXYL1403 and GXNN1839) show novel recombination patterns. GXNN1839 is a recombinant virus that originated from recombination events that occurred in nsp9 and GP2 between strains of lineages 1 (NADC30-like) and 5 (VR2332-like). Many recombinant PRRSV strains that originated from recombination events between NADC30-like and VR2332-like strains have also been identified in previous studies (23, 32, 38, 40). Indeed, the NADC30-like strains seem to be prone to recombine with other PRRSV strains. Numerous recombinant PRRSV strains originated from recombination events between NADC30-like strains and other lineages of PRRSV strains that were found in the field (14, 23, 24, 34, 40–42). GXYL1403 was detected as a recombinant virus from strains of sublineages 8.1 (Ch-1a-like) and 8.3 (JX1A-like) that have circulated in China in recent years. Recombinant PRRSV strains that originated from recombination events that occurred in nsp9 between sublineages 8.1 (Ch-1a-like) and 8.3 (JX1A-like) are also reported in a previous study (42). The emergence of new PRRSV recombination variants poses a major obstacle to the effective control of the spread of virus infection and complicates vaccine development for adequate preventative measures (43). The exact mechanism of genetic recombination in PRRSV remains unknown. The breakpoints of recombination sites in the genome among different PRRS strains can be found at each region of the PRRSV genome and seem to appear randomly. A recently published study shows that nsp9 and GP2–GP3 are the hot spots for PRRSV RNA recombination (44). In this study, we find the two strains (GXYL1403 and GXNN1839) both display a breakpoint in nsp9, and GXNN1839 has an additional breakpoint in GP2, indicating that PRRSV gains genetic diversity through the frequency of recombination events at specific regions with PRRSV strains located in China.

In summary, we determined the complete genome sequences of two novel PRRSV isolates (GXYL1403 and GXNN1839). Both of these are natural recombinant strains that contain amino acid deletions in nsp2. Our findings suggest that PRRSV strains circulating in southern China have undergone recombination among the different lineages or sublineages of field strains, and RNA recombination contributes to their genetic diversity.

## Data Availability Statement

The datasets generated for this study can be found in the nucleotide sequences generated in present study were submitted to the NCBI GenBank with accession numbers, MN660069 and MN660070.

## Author Contributions

ZW, TL, and WH conceived and designed the experiments. JW, SL, DQ, WH, and JH performed the experiments. TR, YC, and KO analyzed the data. JW wrote the manuscript. ZW and TL revised the manuscript. All the authors read and approved the final version of the manuscript.

## Conflict of Interest

The authors declare that the research was conducted in the absence of any commercial or financial relationships that could be construed as a potential conflict of interest.
